# Steroidal response following intravenous administration of long-term frozen tetracosactide acetate in healthy Beagles

**DOI:** 10.1093/jvimsj/aalag124

**Published:** 2026-06-18

**Authors:** Mathilde Vilcot, Nathan Docquier, Anaïs Blazy, Dominique Peeters, Joy Ledeck, Caroline Le Goff, Etienne Cavalier, Elodie Roels

**Affiliations:** Department of Companion Animal Teaching and Clinical Practice, FARAH - Comparative Veterinary Medicine, Faculty of Veterinary Medicine, University of Liège, 4000 Liège, Belgium; Department of Companion Animal Teaching and Clinical Practice, FARAH - Comparative Veterinary Medicine, Faculty of Veterinary Medicine, University of Liège, 4000 Liège, Belgium; Department of Companion Animal Teaching and Clinical Practice, FARAH - Comparative Veterinary Medicine, Faculty of Veterinary Medicine, University of Liège, 4000 Liège, Belgium; Department of Companion Animal Teaching and Clinical Practice, FARAH - Comparative Veterinary Medicine, Faculty of Veterinary Medicine, University of Liège, 4000 Liège, Belgium; Department of Equine Clinical Sciences, Faculty of Veterinary Medicine, FARAH, University of Liège, 4000 Liège, Belgium; Department of Clinical Chemistry, CIRM, Faculty of Medicine, University of Liège, 4000 Liège, Belgium; Department of Clinical Chemistry, CIRM, Faculty of Medicine, University of Liège, 4000 Liège, Belgium; Department of Companion Animal Teaching and Clinical Practice, FARAH - Comparative Veterinary Medicine, Faculty of Veterinary Medicine, University of Liège, 4000 Liège, Belgium

**Keywords:** adrenal, adrenocorticotropic hormone, canine, cortisol, dog, hypoadrenocorticism, stimulation test

## Abstract

**Background:**

The adrenocorticotropic hormone (ACTH) stimulation test is used to assess adrenal function. The effects of extended freezing of tetracosactide acetate (TCA), an ACTH analogue, on test performance, remain unexplored.

**Hypothesis/Objectives:**

To determine whether TCA retains its biological activity to induce adrenal steroid production when stored in plastic syringes and frozen at −20 °C for prolonged period.

**Animals:**

Eight adult experimental Beagles divided into 2 groups of 2 males and 2 females each.

**Methods:**

Prospective case-crossover study. Each dog received 5 μg/kg IV of TCA on 2 occasions, 4 weeks apart (period P1–washout–period P2). Group 1 received frozen then fresh TCA; group 2 received fresh then frozen TCA. Pre- and post-administration blood samples were analyzed at both study periods for cortisol and other steroid metabolites using liquid chromatography–tandem mass spectrometry. A general linear mixed model was used with TCA (fresh vs frozen), period, sequence and timing as fixed factors, and dog as random factor.

**Results:**

Frozen TCA stored for a median time of 1.8 years (IQ range 1.5-1.9) induced similar steroid metabolite responses compared with fresh TCA. Median T1 cortisol concentrations were 248 nmol/L (IQ range 231-272) with frozen TCA and 254 nmol/L (IQ range 249-262) with fresh TCA (*P* = .037). Median T1 17-OHP concentrations were 0.73 μg/L (IQ range 0.67-0.85) with frozen TCA 0.79 μg/L (IQ range 0.71-1.17) with fresh TCA (*P* = .09).

**Conclusions and clinical importance:**

Long-term freezing of TCA in plastic syringes offers a cost-effective strategy and practical alternative during supply shortages.

## Introduction

The adrenocorticotropic hormone (ACTH) stimulation test is used to evaluate adrenocortical function in dogs.^1–4^ It is employed in the diagnosis of primary hypoadrenocorticism,^3–5^ spontaneous or iatrogenic hypercortisolism (HC),^1^^,^^6–8^ but also for monitoring therapy in dogs undergoing medical treatment for spontaneous HC.^9–11^ The test involves measuring baseline serum cortisol (T0), followed by intravenous (IV) or intramuscular administration of synthetic ACTH, commonly tetracosactide acetate (TCA).^3^^,^^4^^,^^12^ A second blood sample is collected 60-90 min later (T1) to evaluate the adrenal response through cortisol concentration.^3–5^ In addition to cortisol, measurement of other adrenal steroids such as 17-hydroxyprogesterone (17-OHP) is gaining attention, particularly in evaluating atypical forms of spontaneous HC.^13–16^ Hence, any investigation into ACTH bioactivity should include a broader panel of adrenal steroid metabolites.

In Europe, TCA is available as 1 mL ampoules (Synacthen®, 0.25 mg/mL; Alfasigma, Bologna, Italy) or vials (Cosacthen®, 0.25 mg/mL; Dechra Veterinary Products SAS, Montigny-Le-Bretonneux, France), intended for immediate use after opening. However, most dogs, especially those weighing <20 kg, require only a small fraction of a vial per ACTH stimulation test (eg, 5 μg/kg for a 15 kg dog = 0.3 mL), resulting in substantial leftover product and increased costs in routine clinical practice. These issues are compounded by occasional supply shortages of TCA depending on the country. To reduce economic losses and mitigate supply challenges, many veterinarians elect to store unused TCA in plastic syringes and freeze it at −20 °C for later use. While the bioactivity of frozen TCA has been confirmed for up to 6 months,^17^ the biological stability of the compound when stored for longer periods remains unexplored. Establishing the efficacy of TCA frozen for an extended period to induce a steroidal response is essential to validate its use in day-to-day practice and avoid compromising diagnostic accuracy.

The aim of this study was to determine whether TCA retains its biological activity after freezing at −20 °C for more than 1 year. We hypothesized that long-term frozen TCA would elicit a comparable adrenal steroid response to freshly prepared TCA in experimental beagle dogs, supporting its clinical use as a cost-effective alternative for routine veterinary endocrinology testing.

## Materials and methods

### Animals and study design

This was a prospective, randomized, case-crossover study conducted at the Small Animal Veterinary Teaching Hospital of the University of Liège (Belgium).

Eight adult, purpose-bred, experimental Beagles were enrolled, with 4 intact males and 4 intact females. None of the dogs showed overt clinical signs of systemic disease at inclusion. All dogs underwent a physical examination to confirm the absence of conditions that could affect the response to the ACTH stimulation test. Throughout the study period, dogs were housed under standard care conditions at the university’s experimental kennel facility. Included dogs did not receive any medication (such as corticosteroids, phenobarbital, ketoconazole,^18^ or trazodone^19^) nor were anesthetized, for a minimum of 2 months before and throughout the study.

**Figure 1:**
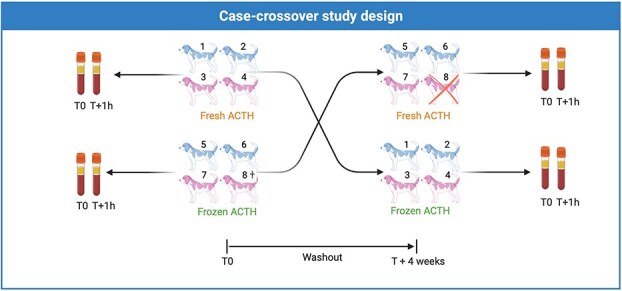
Schematic of the case-crossover design used to study the steroidal response after intravenous administration of long-term frozen tetracosactide acetate (TCA) in experimental Beagles. ^†^Female dog euthanized during the washout period. This dog received only the first TCA injection. Blue dogs were males and pink dogs were females.

Dogs were allocated a number from 1 to 8 and randomized into 2 groups; each group composed of 2 males and 2 females (Figure 1). Included dogs received 2 IV injections of 5 μg/kg of TCA (Synacthen® 0.25 mg/mL; Alfasigma, Bologna, Italy) 4 weeks apart; 1 injection using fresh product from a newly opened vial and the other using product frozen for over a year at −20 °C under routine clinical practice (Figure 1). During the first period (P1) of the study, dogs from group 1 (dogs no. 1, 2, 3, 4) received fresh TCA while dogs from group 2 (dogs no. 5, 6, 7, 8) received long-term frozen TCA. After 4 weeks of wash-out, at period P2, dogs from group 1 received frozen TCA and dogs from group 2 received fresh TCA (Figure 1). At each period, blood samples were collected via jugular venipuncture into dry serum tubes at baseline (T0) and 1 h after TCA injection (T1) (Figure 1); all blood sampling was completed in the morning between 7:30 and 9:30 am. An EDTA-tube and a heparin-tube were also collected at T0 of P1. Dry tubes were centrifuged at 4000 rpm during 15 min at room temperature, and the serum obtained was stored at −80 °C pending batch analysis for steroid measurement at the end of the study protocol. EDTA and heparin blood samples from T0 at P1 were used for hematology (ProCyte Dx, IDEXX Laboratories, Inc., Westbrook, ME, USA) and biochemistry (Catalyst One, IDEXX Laboratories, Inc., Westbrook, ME, USA).

### TCA storage and allocation

Fresh TCA was supplied in 1 mL single-use glass vials (Synacthen® 0.25 mg/mL; Alfasigma, Bologna, Italy). Frozen TCA was obtained from 8 plastic syringes filled with leftover TCA (Synacthen® 0.25 mg/mL; Alfasigma, Bologna, Italy) from previously opened vials stored in the clinic’s freezer. These single-use syringes had been spared over months for later clinical use, labeled with the product name and date of freezing, and stored at −20 °C. The 8 oldest available syringes were selected for the study. Each syringe was thawed immediately before use by warming it in the hands and was randomly assigned to a dog (1 syringe per dog).

### Steroid metabolites analysis

Serum cortisol and the following adrenal steroid metabolites: androstenedione (AND), 17-OHP, dihydrotestosterone (DHT), estradiol (E2), testosterone (TST), progesterone (PROG), sulfated dehydroepiandrosterone (SDHEA), dehydroepiandrosterone (DHEA), 11-deoxycorticosterone (11-DOC), aldosterone (ALDO), cortisone, corticosterone, 11-deoxycortisol (11-S), and 21-deoxycortisol (21-S) were quantified by liquid chromatography–tandem mass spectrometry (LC–MS/MS; LC-30AD and SIL-30AMCP, Shimadzu, Kyoto, Japan; QTRAP 6500, Sciex, Toronto, Canada) at the “Masked for review” laboratory.

### Statistical analysis

Statistical analyses were performed using commercially available software (SAS Version 9.4 for Windows (SAS Institute, Cary, North Carolina, USA)). To assess the equivalence between frozen and fresh TCA to induce a steroidal response, a general linear mixed model was applied to hormonal values obtained at T1. The model included TCA type (fresh vs. frozen), period (P1 vs. P2), sequence (fresh→frozen vs. frozen→fresh), and baseline T0 values as fixed effects, with dog included as a random effect. Variables that were not normally distributed were log-transformed before statistical analysis. Undetectable measurements were replaced with a random value between zero and the lowest detectable concentration, as commonly recommended for steroid data. In addition, paired Student’s *t*-tests were used to compare pre- and post-stimulation mean hormone concentrations (T1 vs. T0), as well as the absolute change in concentration (δ: T1-T0), for each TCA formulation (fresh or frozen) independently. Hormone concentrations at T0 and T1 and the corresponding δ values were illustrated using grouped bar charts.

In addition, an exploratory bioequivalence analysis was performed for cortisol and 17-OHP responses to fresh versus frozen TCA formulations. For each dog at each period, the area under the concentration-time curve (AUC) and maximal concentration (Cmax) were calculated from hormone concentrations measured at T0 and T1. Log-transformed AUC and Cmax values were analyzed using a mixed-effects model including sequence, period, and treatment as fixed effects and dog as random effect. Geometric mean ratios (fresh vs. frozen) and their 90% confidence intervals (90% CI) were estimated. Bioequivalence was assessed using the limits of 0.80-1.25, corresponding to a maximum tolerated variation of 20% around a ratio of 1.^20^

Results are presented as median and interquartile (IQ) range for continuous variables, and as number for qualitative variables. Given the number of hormones evaluated, a more stringent significance threshold was applied to account for multiple testing. Accordingly, a Bonferroni-adjusted significance level of α = 0.0033 (0.05/15) was used, and P-values below this threshold were considered statistically significant.

### Sample size calculation

As the initial hypothesis was that long-term frozen TCA would elicit a comparable cortisol response to freshly prepared TCA (equivalence study), a preliminary sample size calculation was performed using data from a published study,^21^ which assessed biological variability in serum cortisol concentrations after ACTH stimulation in healthy dogs. In that study, the critical difference between sequential post-stimulation cortisol measurements was determined to be 3.3 μg/dL, meaning that any difference greater than this threshold would be considered substantial and unlikely to result from normal biological or analytical variability.^21^ The reported mean (±SD) cortisol concentration after ACTH stimulation was 13.2 ± 1.9 μg/dL.^21^ Using these values (SD = 1.9 μg/dL and equivalence margin d = 3.3 μg/dL), and assuming a paired design (each dog serving as its own control), a sample size of 8 dogs was estimated to provide 90% power to detect a meaningful difference in post-stimulation cortisol concentrations between fresh and frozen TCA administrations.

## Results

### Animals

Eight purpose-bred experimental Beagles (4 males, 4 females) were enrolled in the study, with a median age of 8.9 years (IQ range 7.0-10.9) and a median weight of 14.5 kg (12.0-15.5). Physical examination revealed a mammary mass (fifth right mammary gland) and left inguinal hernia in dog no. 4, keratoconjunctivitis sicca and atopic dermatitis in dog no. 7, and bilateral mammary mass (fifth right and left mammary glands) in dog no. 8; it was unremarkable in all other dogs upon enrollment. Abnormalities were not detected on routine bloodwork at enrollment in all dogs except no. 8, whose biochemistry revealed moderate azotemia (Tables S1 and S2). This dog presented with acute anorexia, lethargy, hematemesis, and melena 19 days after the first TCA injection. Bloodwork showed mild worsening of the azotemia (from 176 to 190 $\mathrm{\mu}$mol/L), and a gastric ulcer was suspected on abdominal ultrasound. The dog was subsequently euthanized in accordance with the experimental kennel’s policy. Post-mortem examination revealed multiple deep gastric ulcers and advanced bilateral chronic kidney disease. The decision to euthanize was unrelated to the ongoing research protocol. This dog, an intact female, had received only the first injection of TCA using frozen TCA, resulting a slightly imbalanced group composition at P2.

### TCA administration

The median volume of TCA administered per dog was 0.29 mL (0.24-0.31). Frozen TCA was stored for a median of 22.1 months (17.8-23.0) before administration, with a maximum length of storage of 24.6 months (2.05 years).

### TCA bioactivity

Steroid hormone concentration data obtained for each dog at each time point and in each period are presented in Table S3; with undetectable values highlighted in red and values below the lower limit of quantification (LOQ) highlighted in orange for each hormone.

### Cortisol

**Figure 2:**
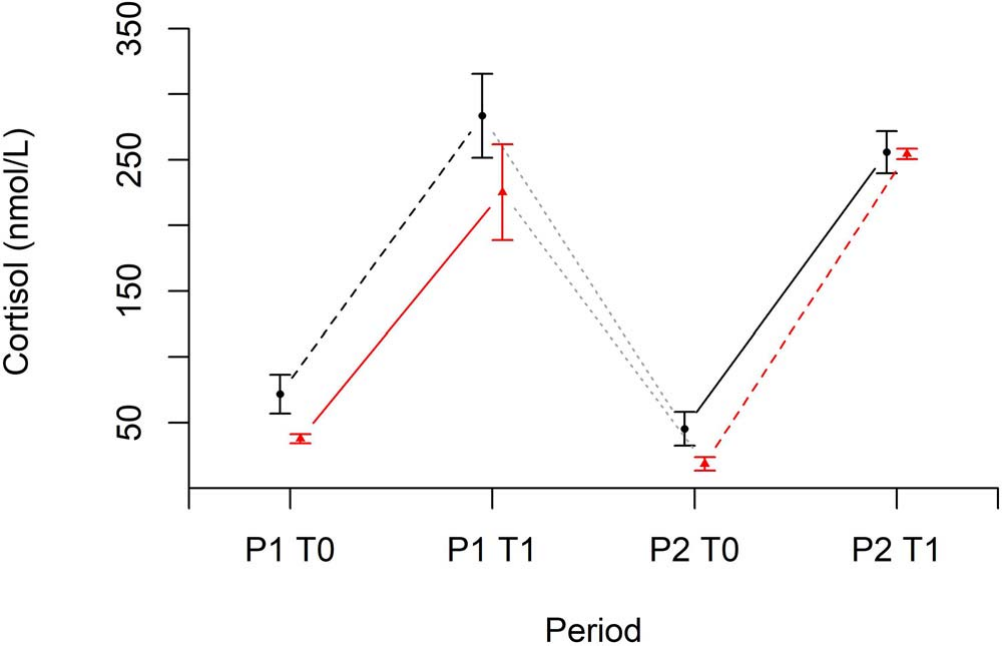
Mean serum cortisol concentrations (nmol/L) before (T0) and 1 h after (T1) intravenous administration of tetracosactide acetate (TCA) for each group, sequence and period in experimental Beagles. Sequences are represented in black (fresh→frozen) or red (frozen→fresh); fresh TCA in dash line and frozen TCA in solid line; wash-out is in dotted line. Points represent mean values and whiskers indicate standard error. Fresh TCA induced a significant increase in cortisol concentration with a δ T1-T0 of 226.3 (185.74-251.28) nmol/L (*P* < .0001). The same was observed for frozen TCA with a δ T1-T0 of 213.63 (178.46-234.84) nmol/L (*P* < .0001).

There was no effect of the TCA type (fresh vs. frozen) on T1 cortisol concentration (*P* = .37), indicating that fresh and frozen TCA induced similar cortisol responses. Median cortisol concentrations were 50.7 nmol/L (17.6-69.7) at T0 and 254.3 nmol/L (249-261.5) at T1 with fresh TCA, and 37.2 nmol/L (29.6-49.5) at T0 and 248.3 nmol/L (231.5-272.0) at T1 with long-term frozen TCA. These results are illustrated in Figure 2. There was no effect of the baseline T0 values, sequence, or period for cortisol concentrations obtained at T1.

Additionally, the mean geometric ratio was 0.95 (90% CI: 0.85-1.07) for the AUC and 0.96 (90% CI: 0.90-1.03) for Cmax, supporting bioequivalence between fresh and frozen TCA for stimulation of cortisol production.

### 17-OHP

**Figure 3:**
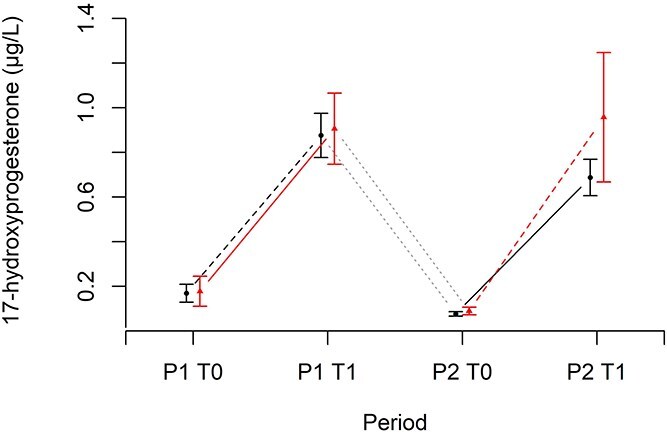
Mean serum 17-OHP concentrations (μg/L) before (T0) and 1 h after (T1) intravenous administration of tetracosactide acetate (TCA) for each group, sequence and period in experimental Beagles. Sequences are represented in black (fresh→frozen) or red (frozen→fresh); fresh TCA in dash line and frozen TCA in solid line; wash-out is in dotted line. Points represent mean values and whiskers indicate standard error. Fresh TCA induced a significant increase in 17-OHP concentration with a δ T1-T0 of 0.59 (0.56-0.99) μg/L (*P* = .0009). The same was observed for frozen TCA with a δ T1-T0 of 0.64 (0.48-0.76) μg/L (*P* = .0003).

There was no effect of the TCA type (fresh vs. frozen) on T1 17-OHP concentration (*P* = .09). Median 17-OHP concentrations were 0.12 μg/L (0.06-0.22) at T0 and 0.79 μg/L (0.71-1.17) at T1 with fresh TCA, and 0.09 μg/L (0.08-0.13) at T0 and 0.73 μg/L (0.67-0.85) at T1 with long-term frozen TCA. These results are illustrated in Figure 3. There was no effect of the baseline T0 values, sequence, or period for 17-OHP concentrations obtained at T1.

The mean geometric ratio was 0.88 (90% CI: 0.81-0.96) for the AUC and 0.91 (90% CI: 0.83-0.99) for Cmax, supporting bioequivalence between fresh and frozen TCA for stimulation of 17-OHP production.

### Other steroid hormones

Detailed analyses for the 13 steroid hormones evaluated in addition to cortisol and 17-OHP are provided in Supplementary Data 4.

## Discussion

This study demonstrated that TCA stored in plastic syringes and frozen at −20 °C for up to 2 years retains its biological activity and elicits adrenal steroid responses comparable to that of freshly prepared TCA. No significant difference was observed in post-stimulation cortisol and 17-OHP concentrations or in the response of the other adrenal steroid hormones studied between fresh and long-term frozen TCA.

The ACTH stimulation test is a cornerstone in the diagnosis and monitoring of adrenal disorders in dogs.^1^^,^^4^^,^^5^^,^^8^ The present study showed no significant difference in cortisol concentrations at 1-h post-stimulation (T1) between frozen and fresh TCA, and both produced a robust and significant cortisol response compared with baseline (T0), in accordance with established reference data for healthy dogs undergoing ACTH stimulation.^21^ Bioequivalence analysis further confirmed these findings, with both AUC and Cmax largely within the standard bioequivalence range of 0.8-1.25, indicating similar adrenal stimulation by fresh and frozen TCA. In our study, the maximal cortisol concentration (Cmax) coincided with T1 for all dogs. These results align with earlier findings that reported comparable cortisol responses after administration of frozen cosyntropin over shorter durations (up to 6 months at −20 °C).^17^ This study confirms preserved biological activity after extended freezing storage at −20 °C for up to 2 years.

The use of LC–MS/MS allowed simultaneous measurement of multiple adrenal steroids, providing a broader evaluation of TCA bioactivity. TCA administration induced a significant increase in 17-OHP, PROG, 11-DOC, ALDO, cortisone, corticosterone, 11-S, and 21-S levels at T1; whereas there was no stimulatory effect for the other measured hormones including AND, DHT, E2, TST, and DHEA, as has been previously shown^22^^,^^23^ in intact dogs. Among these, 17-OHP was of particular interest, as it plays a key role in the evaluation of atypical spontaneous HC.^13–16^^,^^24^ Of importance, the 17-OHP concentrations increase after TCA stimulation was unaffected by TCA storage status. This consistent response underscores the validity of long-term frozen TCA in dynamic testing protocols for evaluation of atypical spontaneous HC.^14^^,^^16^^,^^24^^,^^25^

An effect of baseline hormonal values (T0) on post-stimulation values (T1) was observed for some steroids, including AND, DHT, TST, PROG, and DHEA. Concentrations obtained at T1 for these hormones were higher if the values before stimulation were high. This suggests that pre-stimulation adrenal activity strongly influences the magnitude of the steroidogenic response to TCA stimulation. This pattern echoes previous findings in healthy dogs.^22^^,^^23^^,^^26^

An exploratory period effect was observed for 17-OHP, with higher concentrations in P1 compared with P2 regardless of treatment group. However, this effect was no longer significant after Bonferroni correction for multiplicity. The most plausible explanation for this difference is the loss of one female dog between the 2 periods, resulting in a slightly unbalanced sex ratio in Period 2 (3 females vs 4 males). As all dogs included were intact and hormonally active, this change in group composition could have influenced the median 17-OHP concentrations, as some studies suggested higher 17-OHP concentrations in females compared with males.^27^ Differences in the stage of the estrous cycle among females could also have contributed to this variability. 17-OHP concentrations fluctuate throughout the canine reproductive cycle, with higher values reported during estrus and diestrus compared with anestrus.^28^ Seasonal or circadian influences have been described.^16^^,^^22^^,^^23^ However, since all tests were performed at the same time of day and during the same season for all animals, these variations are unlikely to explain the observed effects. Notably, TCA type (fresh vs. frozen) had no significant impact on 17-OHP concentrations obtained after stimulation at T1, reinforcing the overall equivalence of fresh and frozen TCA to induce steroidal response.

This study has some limitations. First, the small sample size, although statistically powered for cortisol,^21^ might limit detection of smaller differences for other steroidal hormones. Additionally, one dog was euthanized between periods for a reason unrelated to the research project, resulting in missing data for the second TCA stimulation test in that individual. This would have slightly reduced statistical power. Furthermore, cortisol and other steroids concentrations were evaluated at a single post-stimulation timepoint (60 min). Although this timing is standard for cortisol measurement, other adrenal steroids might peak at different intervals, and differences could have emerged at alternative sampling times.^22^ Additionally, the estrus status of the female dogs was not assessed by vaginal smear cytology, and potential effects of reproductive stage on sex hormones concentrations cannot be excluded. Finally, as this study was conducted on purpose-bred experimental dogs, this might not fully reflect the endocrine response of dogs with naturally occurring adrenal disease.

In conclusion, long-term freezing of TCA for up to 2 years does not diminish its biological effectiveness in stimulating adrenal steroidogenesis, particularly cortisol and 17-OHP production, in experimental dogs. This approach offers a practical and cost-effective strategy for routine endocrine testing, particularly during periods of supply shortages or for clinics managing partial vial wastage.

## Supplementary Material

Supplementary_material_aalag124

## Off-label antimicrobial declaration

Authors declare no off-label use of antimicrobials.

## Institutional animal care and use committee or other approval declaration

Approved by the Institutional Animal Care and Use Committee from the University of Liège, project identification: 23-2610, category and retrospective evaluation: C, project classified as an experiment.

## Abbreviations

11-DOC: 11-deoxycorticosterone
11-S: 11-deoxycortisol
17-OHP: 17-hydroxyprogesterone
21-S: 21-deoxycortisol
ACTH: adrenocorticotropic hormone
ALDO: aldosterone
AND: androstenedione
AUC: Area under the curve
Cmax: maximal concentration
DHEA: dehydroepiandrosterone
DHT: dihydrotestosterone
E2: estradiol
GLMM: general linear mixed model
HC: hypercortisolism
IV: intravenous
LC–MS/MS: liquid chromatography–tandem mass spectrometry
LOQ: limit of quantification
PROG: progesterone
SDHEA: sulfate dehydroepiandrosterone
TCA: tetracosactide acetate
TST: testosterone
