# Supplementary material for: Steroidal response following intravenous administration of long-term frozen tetracosactide acetate in healthy Beagles
Source: J Vet Intern Med. 2026 Jun 18;40(3):aalag124. doi: 10.1093/jvimsj/aalag124 (PMC13278770; doi:10.1093/jvimsj/aalag124)

**Supplementary Data 4.** TCA bioactivity for other steroid hormones.

Among the 13 steroid hormones evaluated in addition to cortisol and 17-OHP, five showed undetectable baseline measurements (T0) in some dogs: DHT (in 2 females at all periods and in 1 female at P1), E2 (in 1 female at all period, and in 1 female and 2 males at P2), ALDO (in 3 females and 2 males at all periods, and in 1 male at P2), 21-S (in 2 females and 1 male at all periods, 1 female at P1, and 1 female and 2 males at P2), and DHEA (in 2 females at all periods, and in 2 females at P1) (Supplementary Table 3). After stimulation (T1), DHT concentrations remained undetectable in all female dogs that had undetectable baseline values, with one additional female showing undetectable concentrations at P2 (Supplementary Table 3). Similarly, E2 remained undetectable after stimulation in all dogs with undetectable baseline concentrations. DHEA remained undetectable after stimulation in two out of the four females with undetectable baseline concentrations (Supplementary Table 3). For SDHEA, all values were undetectable across time points and periods for all included dogs; consequently, no statistical analyses were performed for this steroid metabolite (Supplementary Table 3).

Across the remaining 12 hormones evaluated, no significant effect of TCA type (fresh *vs.* frozen) was observed for post-stimulation (T1) values. As displayed in Supplementary Figure 4 below, both TCA type elicited similar steroid hormone responses; the Delta T1-T0 was significant for PROG, 11-DOC, ALDO, cortisone, corticosterone, 11-S and 21-S at all periods (P<.002).

There was an effect of baseline T0 concentrations observed for several hormones on T1 values, with higher T1 concentrations observed when baseline T0 values were elevated. These included AND (P<.0001), DHT (P<.0001), TST (P<.0001), PROG (P=.001) and DHEA (P<.0001).

No other effects related to TCA type, baseline T0 concentrations, period, or sequence were observed on T1 steroid hormone concentrations.

**Supplementary Figure 4.** Mean serum concentrations of androstenedione, dihydrotestosterone, estradiol, testosterone, progesterone (PROG), sulfate dehydroepiandrosterone, dehydroepiandrosterone, 11-deoxycorticosterone (11-DOC), aldosterone (ALDO), cortisone, corticosterone, 11-deoxycortisol (11-S), and 21-deoxycortisol (21-S) before (T0) and 1 hour after (T1) intravenous administration of tetracosactide acetate (TCA) for each group, sequence and period in experimental Beagles.

Sequences are represented in black (fresh→frozen) or red (frozen→fresh) ; fresh TCA in dash line and frozen TCA in solid line; wash-out is in grey. Points represent mean values and whiskers indicate standard error.

A significant increase from T0 to T1 after TCA administration was observed for PROG (P=.0004), 11-DOC (P=.0002), ALDO (P<.002), cortisone (P=.0009), corticosterone (P=.0002), 11-S (P=.0007), and 21-S (P=.0002) at all periods.


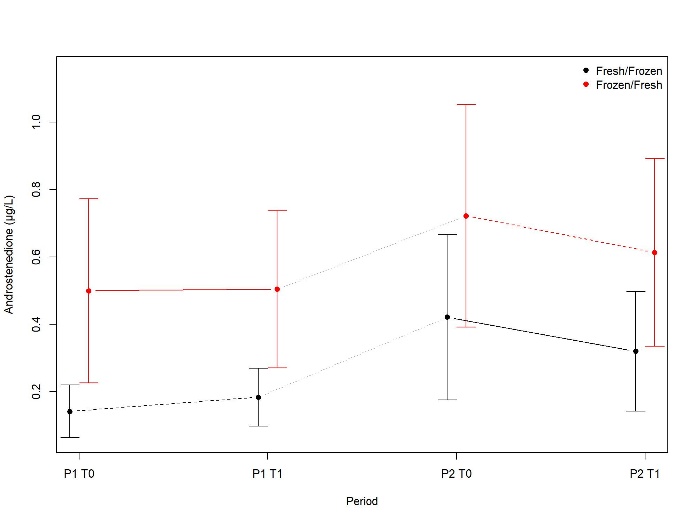

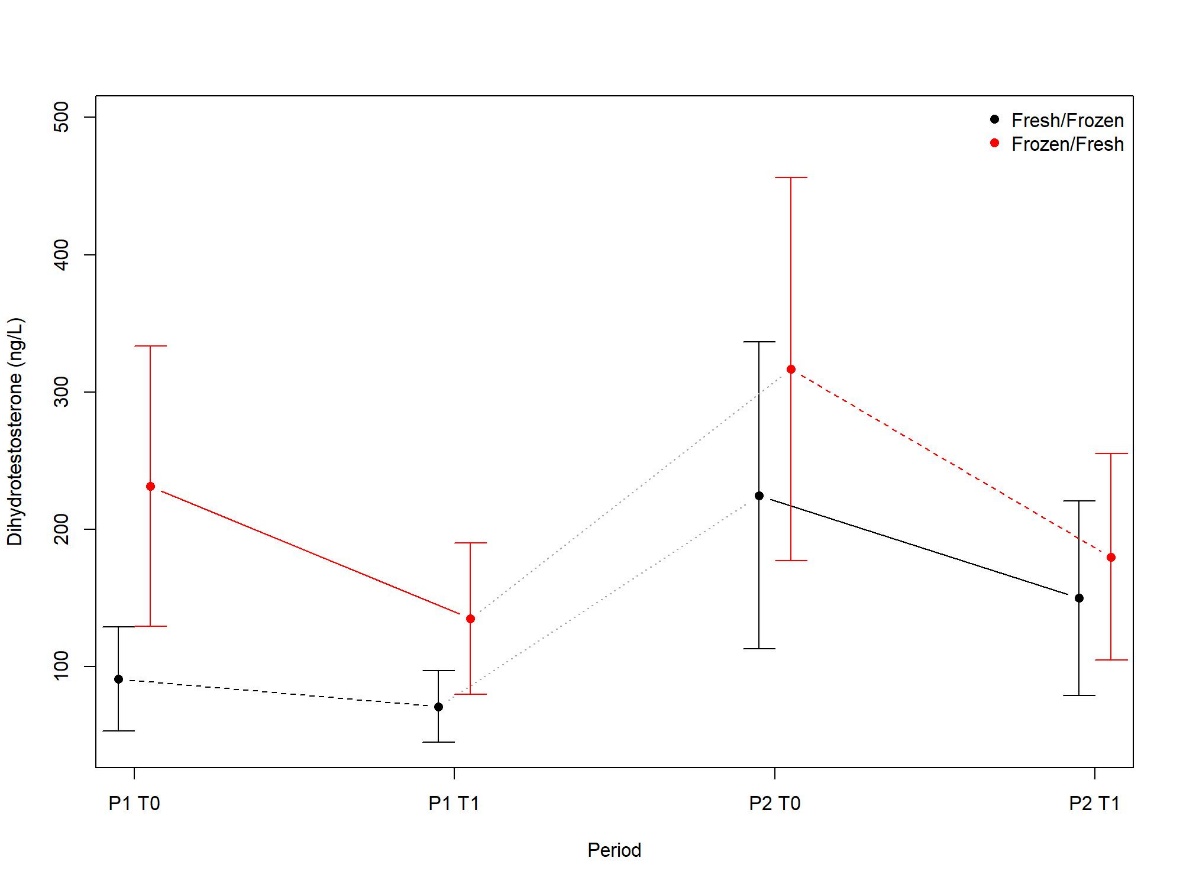


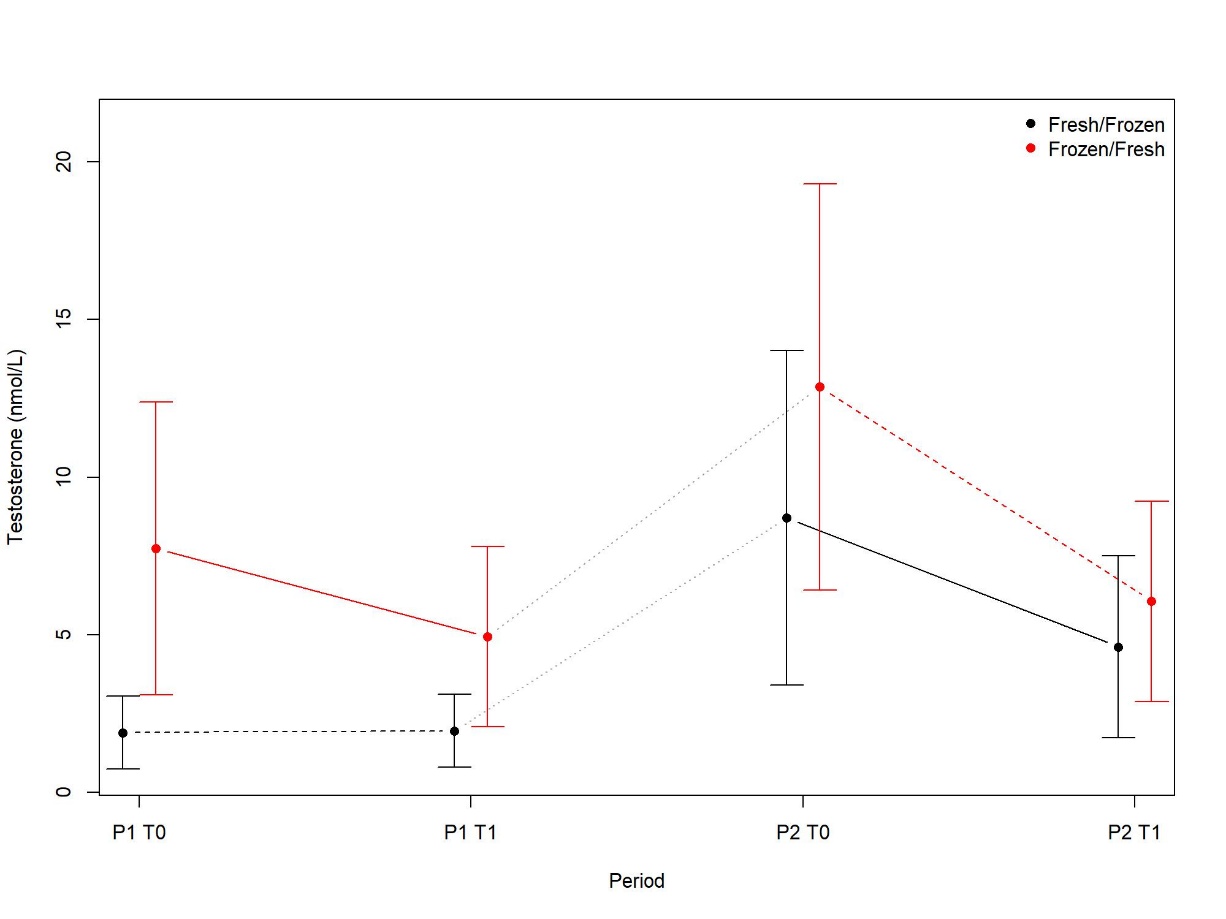

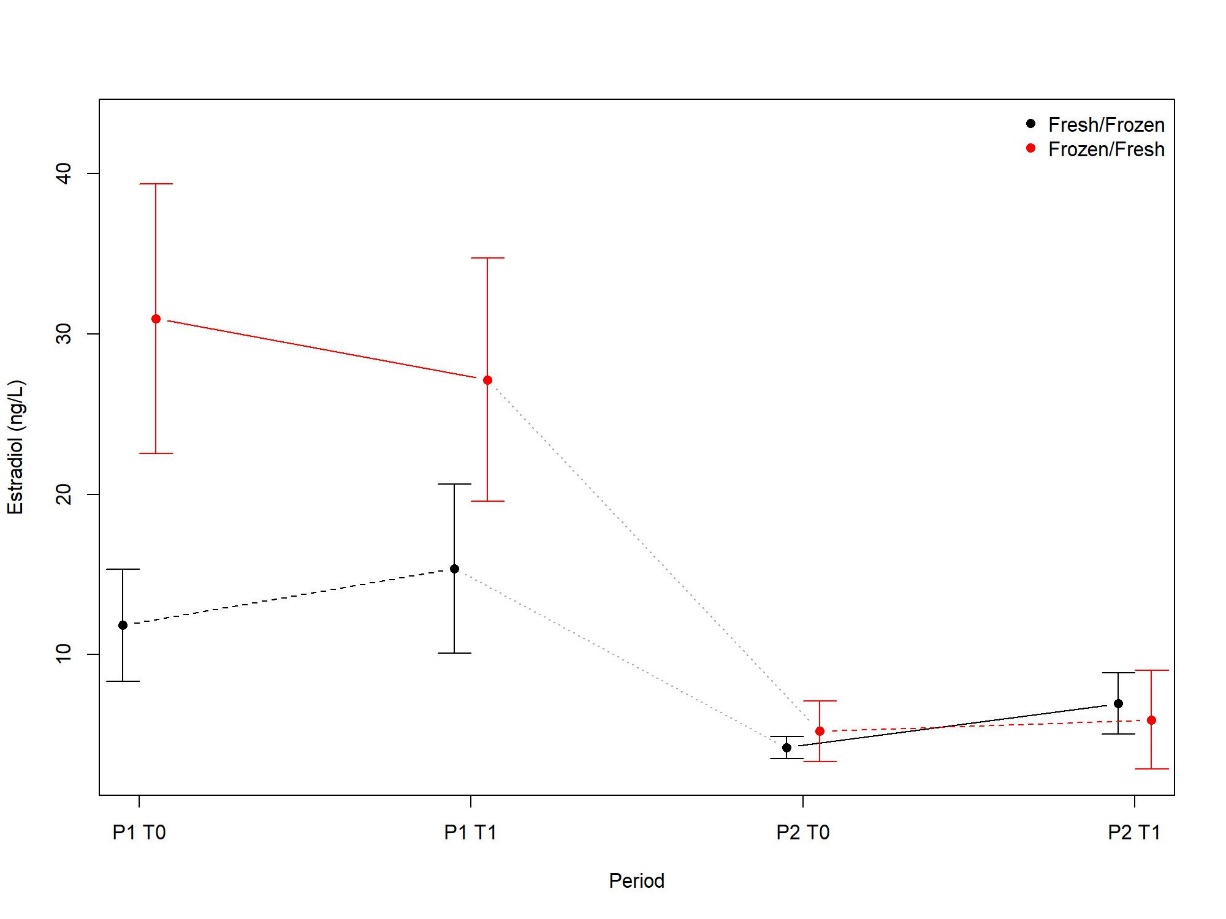


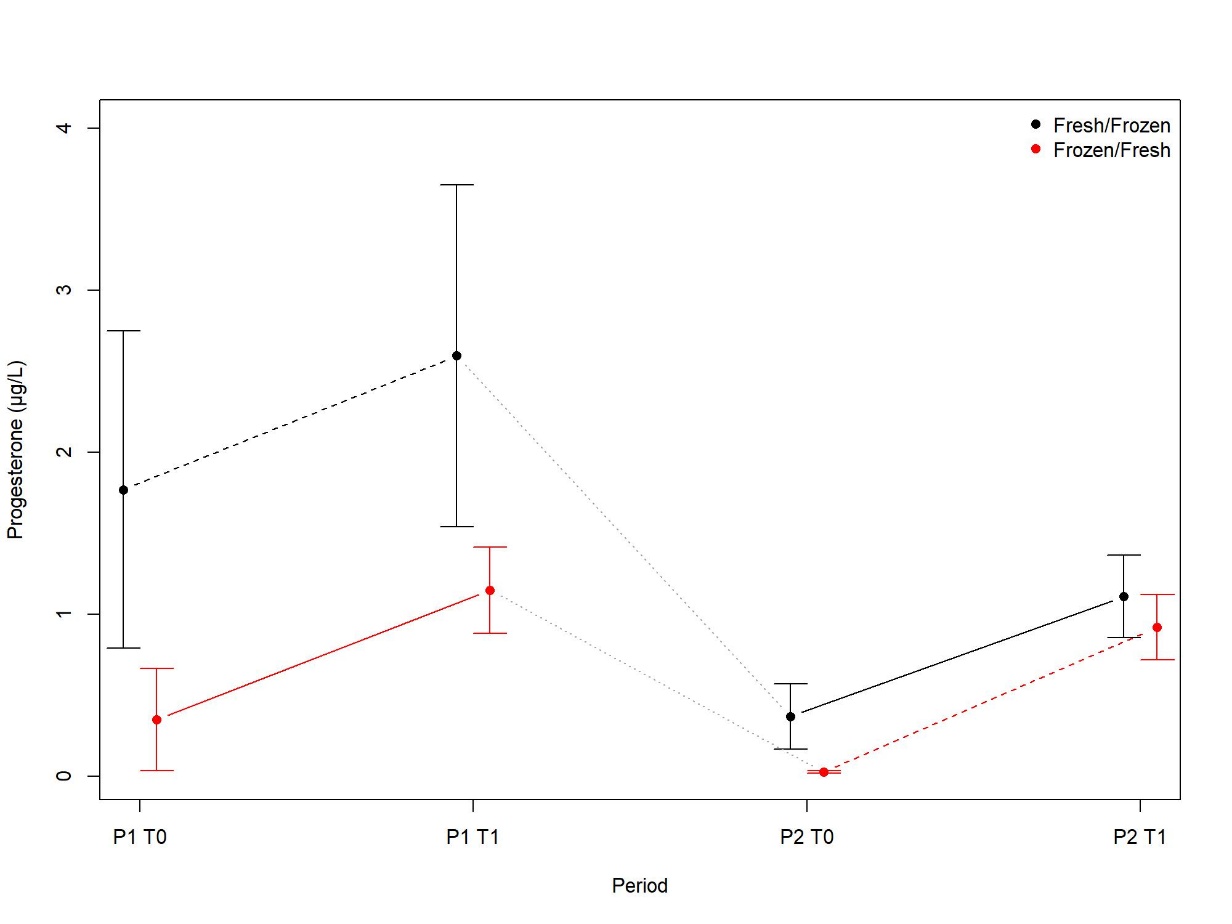

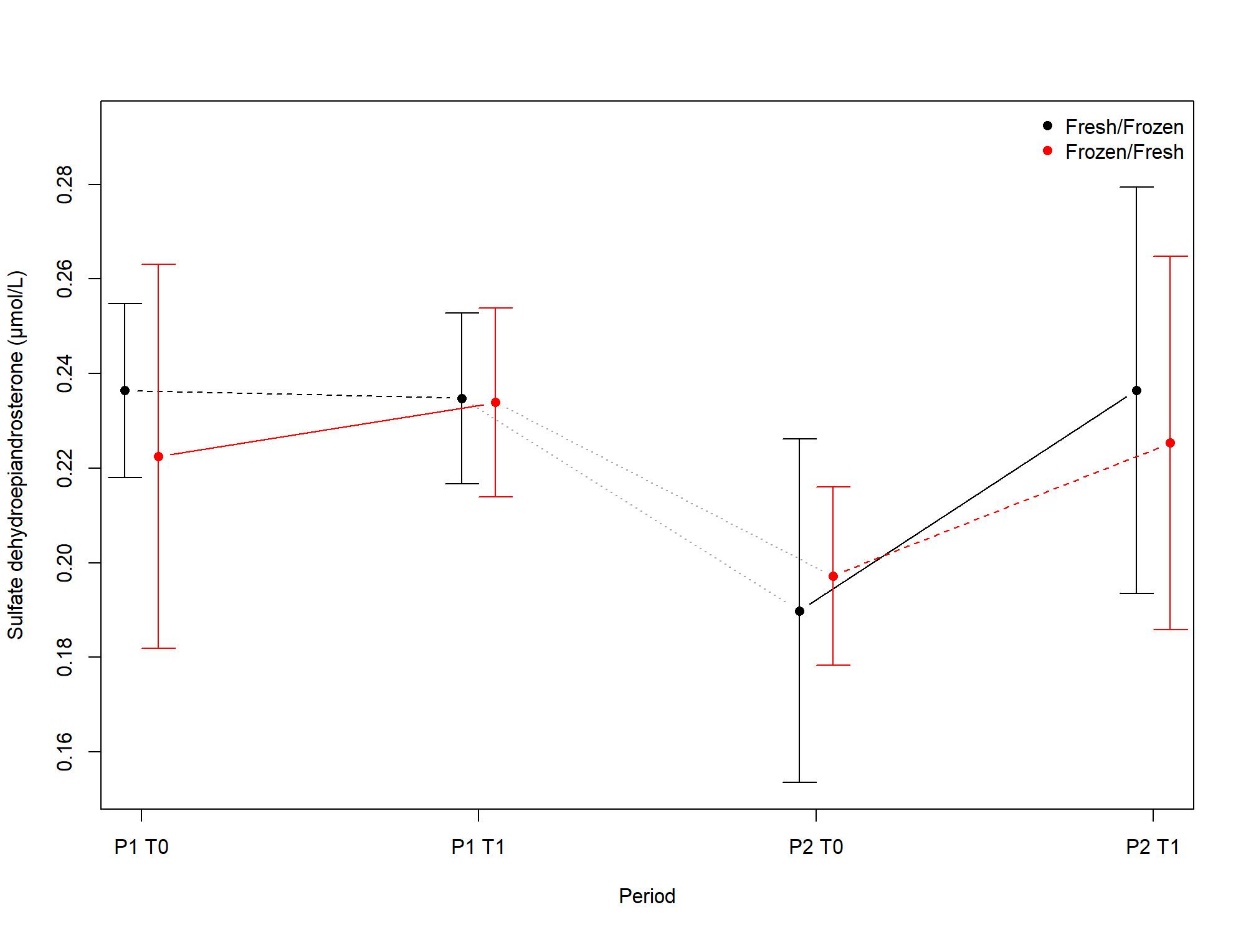


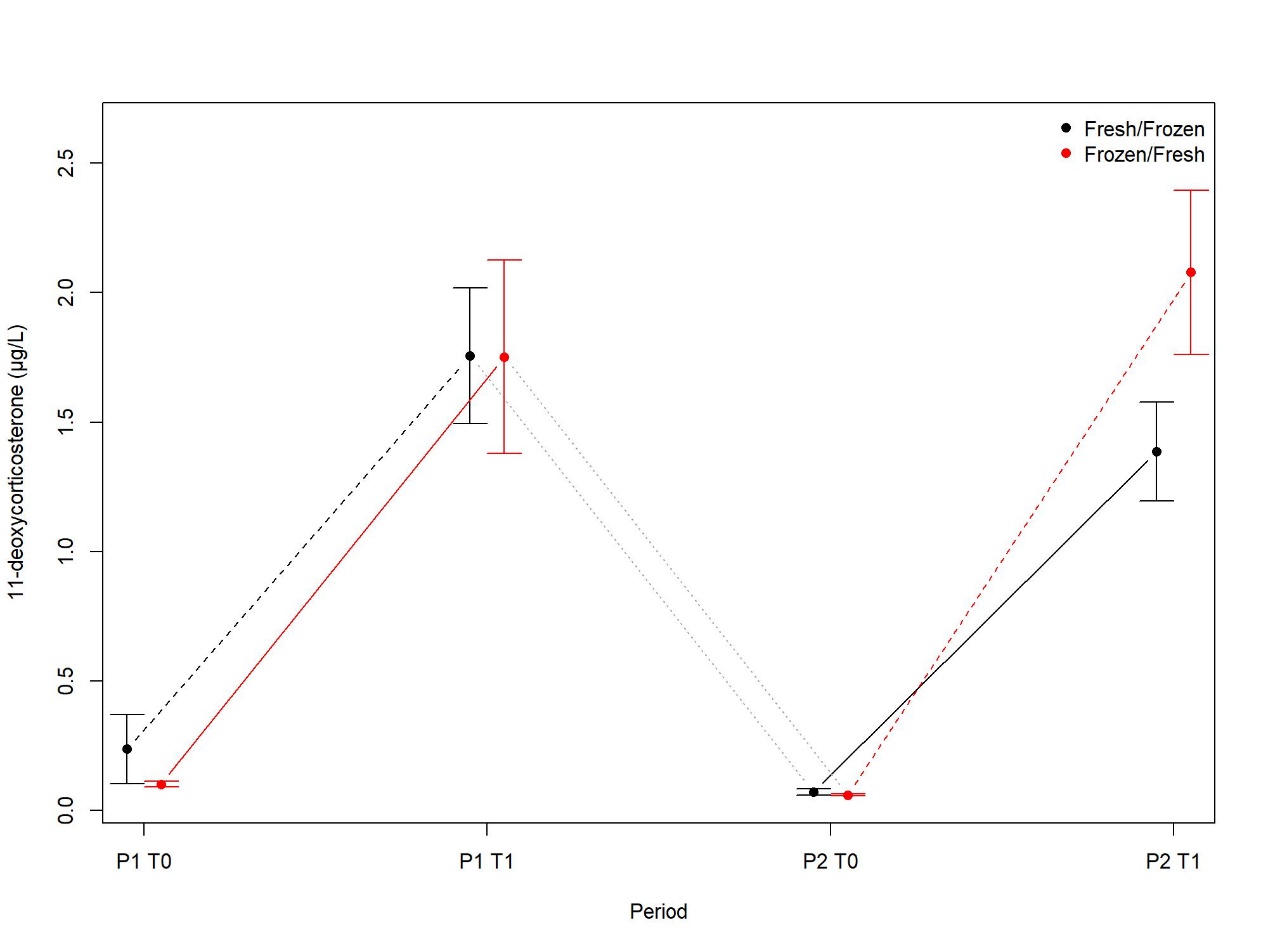

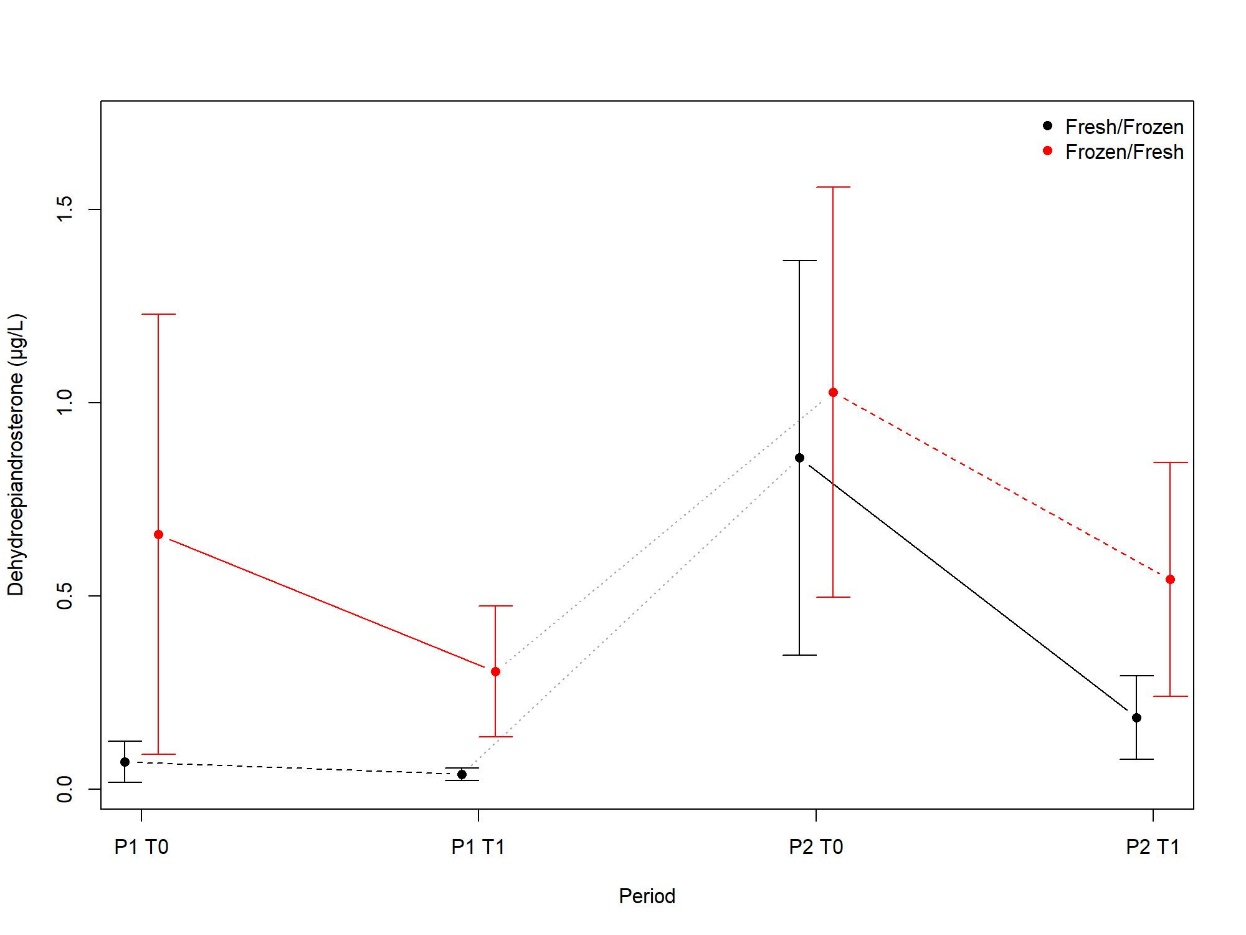


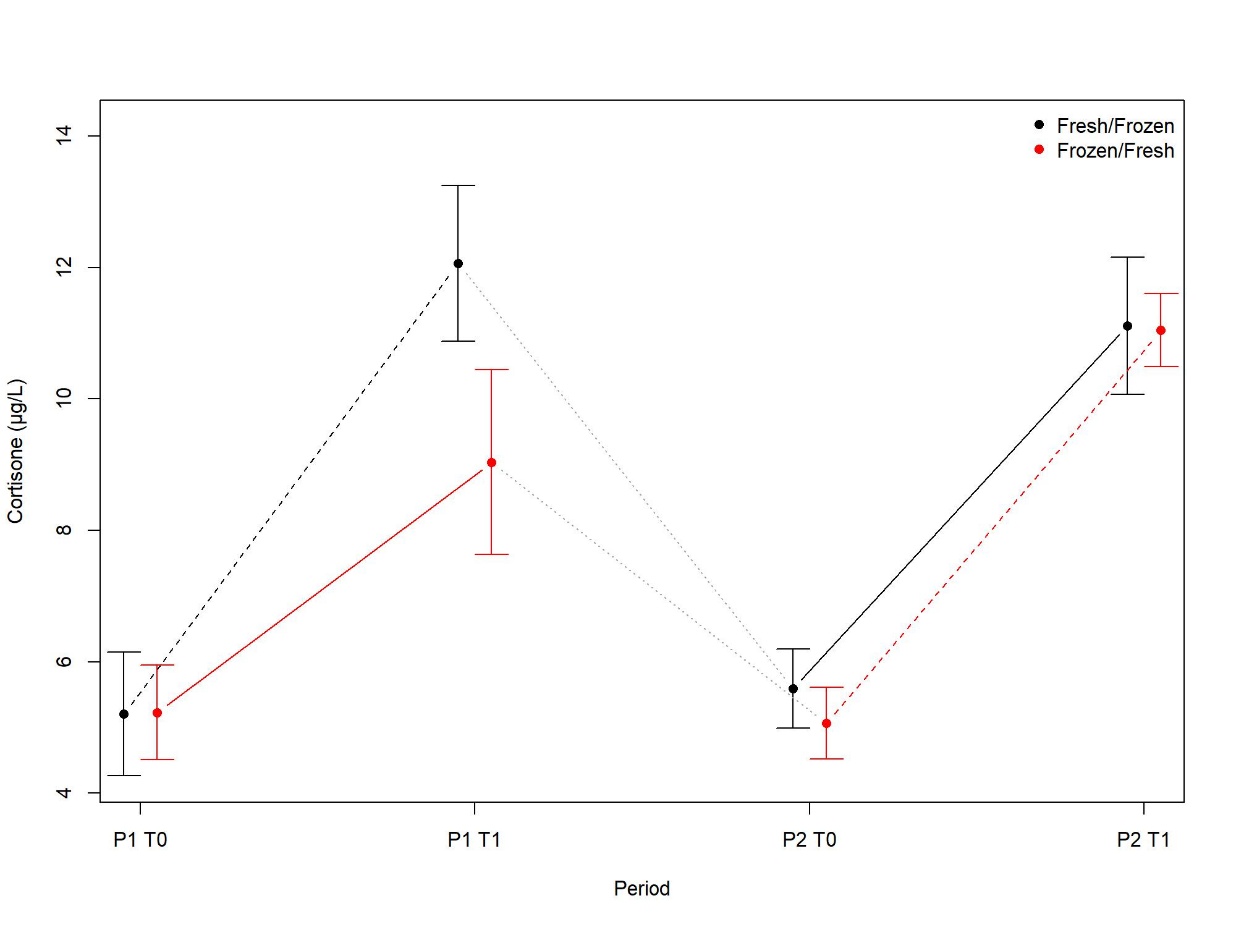

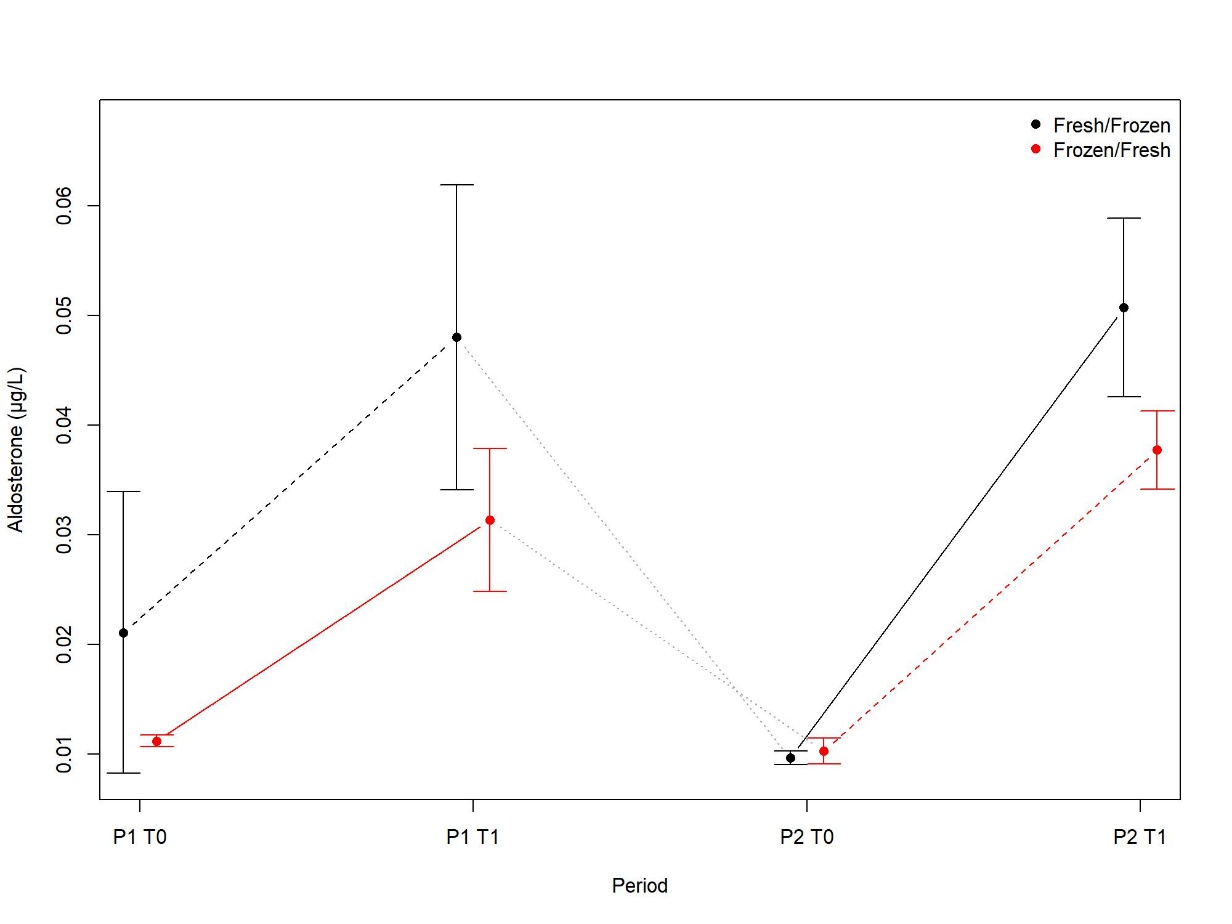


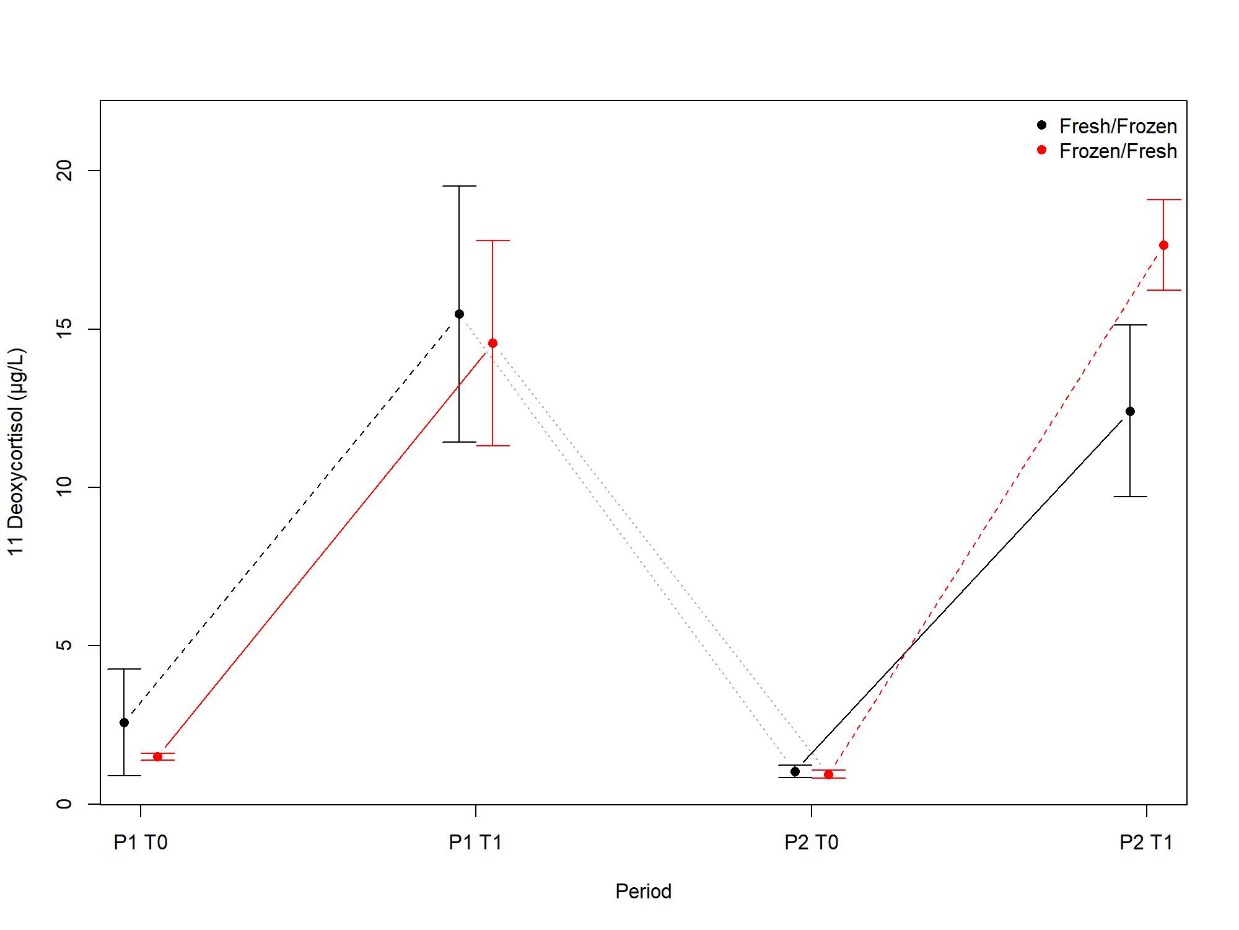

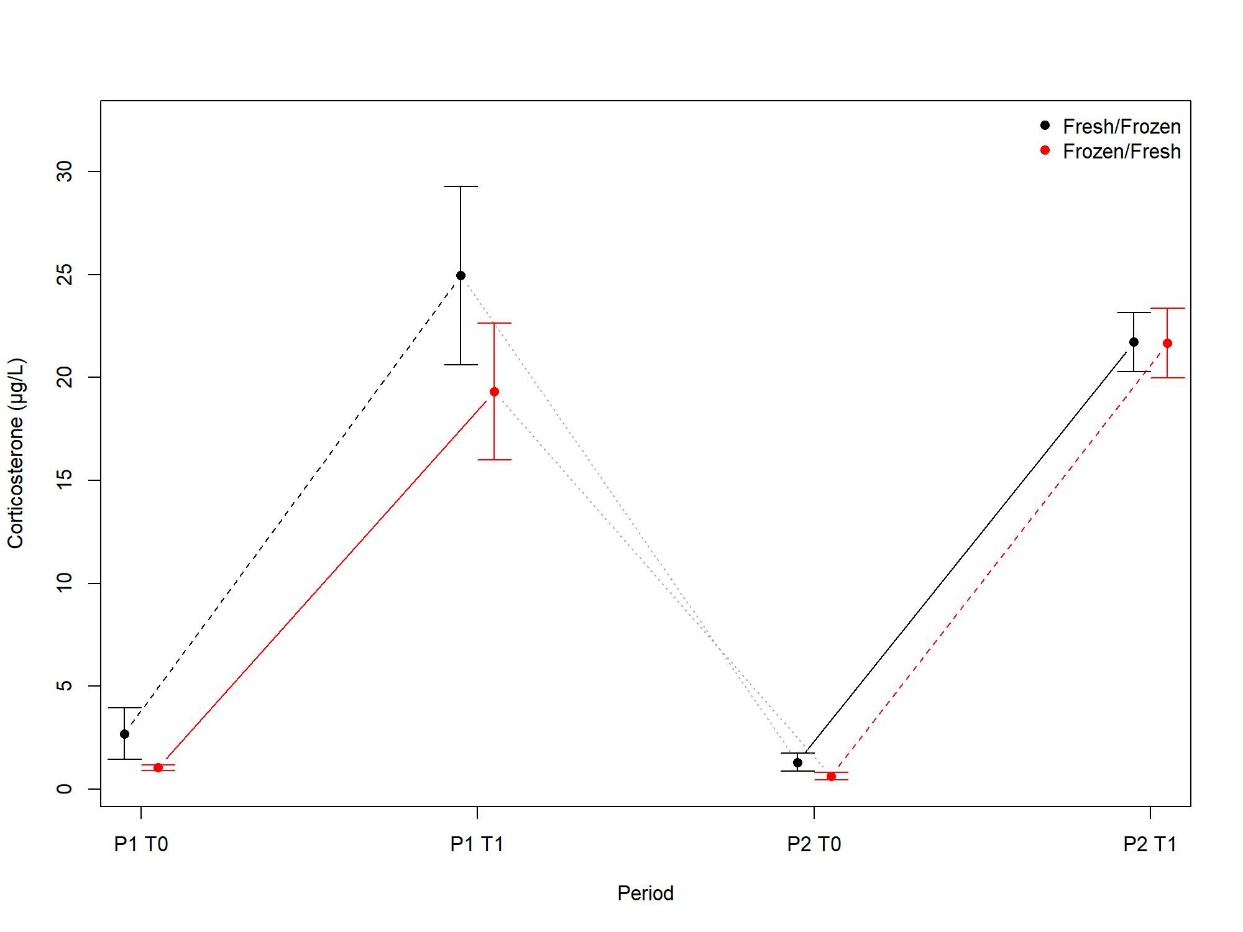


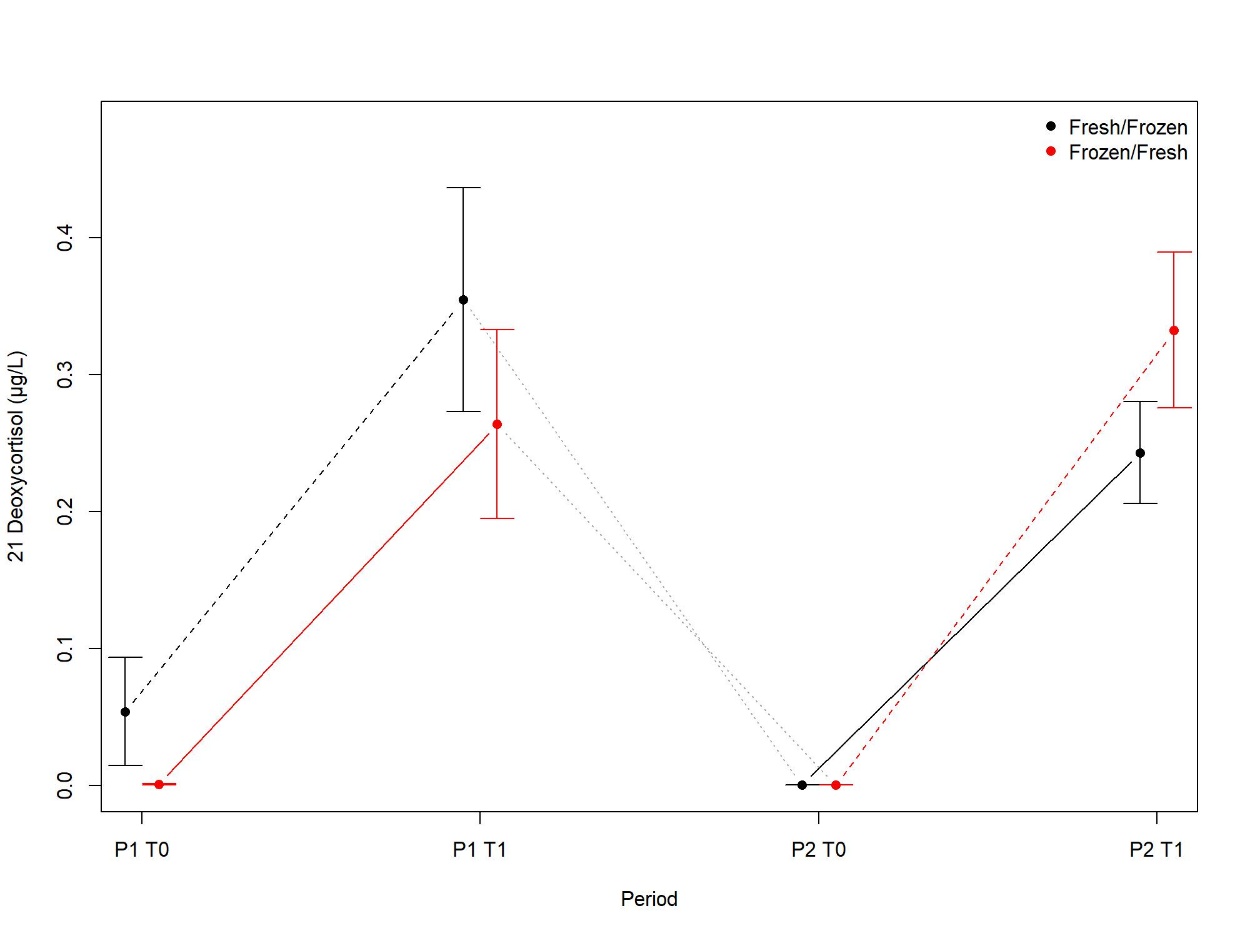

Supplement: Supplementary_material_aalag124 [file supplementary_material_aalag124.zip › Supplementary_data_4_clean.docx]
