# Supplementary material for: Steroidal response following intravenous administration of long-term frozen tetracosactide acetate in healthy Beagles
Source: J Vet Intern Med. 2026 Jun 18;40(3):aalag124. doi: 10.1093/jvimsj/aalag124 (PMC13278770; doi:10.1093/jvimsj/aalag124)
Supplement: Supplementary_material_aalag124 [file supplementary_material_aalag124.zip › Supplementary table 1.docx]

**Supplementary Table 1.** Hematology results of the included Beagles at enrollment.

| **Parameters** | **Dog 1** | **Dog 2** | **Dog 3** | **Dog 4** | **Dog 5** | **Dog 6** | **Dog 7** | **Dog 8** | **Reference range** |
| --- | --- | --- | --- | --- | --- | --- | --- | --- | --- |
| **Red blood cells x10^12^/L** | 8.90 | 8.3 | 6.3 | 6.91 | 7.86 | 8.7 | 6.78 | ***5.18*** | 5.65 – 8.87 |
| **Hematocrit (%)** | 59.6 | 55.6 | 45.1 | 46.8 | 56.1 | 55.9 | 42.8 | ***35.6*** | 37.3 – 61.7 |
| **Hemoglobin (g/L)** | 204 | 189 | 155 | 154 | 194 | 202 | 150 | ***122*** | 131 – 205 |
| **MCV (fL)** | 66.9 | 67.0 | 71.6 | 67.7 | 71.4 | 64.3 | 63.1 | 68.7 | 61.6 – 73.5 |
| **MCH (pg)** | 22.9 | 22.8 | 24.6 | 22.3 | 24.7 | 23.2 | 22.1 | 23.6 | 21.2 – 25.9 |
| **MCHC (g/L)** | 342 | 340 | 344 | 329 | 346 | 361 | 350 | 343 | 320 – 379 |
| **Reticulocytes (K/µL)** | 56.1 | 89.6 | 80.0 | 69.1 | 39.3 | 35.7 | 30.5 | 20.7 | 10 – 110 |
| **Total leucocytes x10^9^/L** | 5.68 | 5.26 | 6.46 | 9.26 | 6.48 | 7.05 | 9.91 | 9.19 | 5.05 – 16.76 |
| **Neutrophils x10^9^/L** | 3.2 | 3.58 | 3.83 | 4.75 | 3.94 | 4.71 | 6.64 | 6.42 | 2.95 – 11.64 |
| **Lymphocytes x10^9^/L** | 1.73 | 1.19 | 2.05 | 3.54 | 1.84 | 1.73 | 2.49 | 1.86 | 1.05 – 5.10 |
| **Monocytes x10^9^/L** | 0.43 | 0.32 | 0.43 | 0.63 | 0.37 | 0.43 | 0.54 | 0.69 | 0.16 – 1.12 |
| **Eosinophils x10^9^/L** | 0.31 | 0.17 | 0.15 | 0.25 | 0.28 | 0.15 | 0.22 | 0.20 | 0.06 – 1.23 |
| **Basophils x10^9^/L** | 0.01 | 0.0 | 0.0 | 0.09 | 0.05 | 0.03 | 0.02 | 0.02 | 0 – 0.10 |
| **Thrombocytes x10^9^/L** | 315 | 286 | ***576*** | 336 | 176 | 294 | 338 | 426 | 148 – 484 |
