# Supplementary material for: Steroidal response following intravenous administration of long-term frozen tetracosactide acetate in healthy Beagles
Source: J Vet Intern Med. 2026 Jun 18;40(3):aalag124. doi: 10.1093/jvimsj/aalag124 (PMC13278770; doi:10.1093/jvimsj/aalag124)
Supplement: Supplementary_material_aalag124 [file supplementary_material_aalag124.zip › Supplementary table 2.docx]

**Supplementary Table 2.** Biochemistry results of the included Beagles at enrollment.

| **Parameters** | **Dog 1** | **Dog 2** | **Dog 3** | **Dog 4** | **Dog 5** | **Dog 6** | **Dog 7** | **Dog 8** | **Reference range** |
| --- | --- | --- | --- | --- | --- | --- | --- | --- | --- |
| **Glucose (mmol/L)** | 5.12 | 5.35 | 5.24 | 5.37 | 5.41 | 5.28 | 5.15 | 4.9 | 3.89 – 7.95 |
| **Creatinine (µmol/L)** | 79 | 71 | 34 | 64 | 66 | 40 | 48 | ***176*** | < 125 |
| **Urea (mmol/L)** | 4.3 | 6.1 | 4.6 | 4.2 | 4.5 | 3.8 | 4.9 | ***23.7*** | 2.5 – 9.6 |
| **Total proteins (g/L)** | 52 | 53 | 60 | 60 | 56 | 60 | 60 | 62 | 52 – 82 |
| **Albumin (g/L)** | 23 | 25 | 28 | 28 | 26 | 28 | 27 | 27 | 23 – 40 |
| **Globulin (g/L)** | 29 | 28 | 32 | 32 | 30 | 32 | 33 | 35 | 25 – 45 |
| **ALT (U/L)** | 125 | 43 | 45 | 54 | 103 | 43 | 81 | 43 | 10 – 125 |
| **ALP (U/L)** | 47 | 35 | 133 | 71 | 57 | 122 | 102 | 158 | 23 – 212 |
| **Na (mmol/L)** | 149 | 150 | 155 | 150 | 153 | 154 | 153 | 153 | 144 – 160 |
| **K (mmol/L)** | 4.2 | 4.0 | 4.5 | 3.4 | 3.7 | 4.5 | 4.1 | 4.7 | 3.5 – 5.8 |
| **Cl (mmol/L)** | 112 | 111 | 111 | 109 | 110 | 110 | 111 | 112 | 109 – 122 |
